# Medical Convention

**Published:** 1867-03

**Authors:** 


### Medical Convention.

At the meeting of the American Medical Association held in the city of Baltimore, May 3d, 1866, the following resolution was adopted with much unanimity, and the undersigned appointed a committee to aid in carrying it into practical effect:

"*Resolved*, That this Association earnestly requests the medical colleges of the country to hold a convention for the purpose of thoroughly revising the present system of medical college instruction, and that a committee be appointed to aid in carrying the resolution into effect."

In fulfilling the duties enjoined on them, the undersigned respectfully and earnestly invite the Trustees and Faculty of each regular medical college in the United States to send representatives to a convention to be held in the city of Cincinnati, Ohio, on Friday preceding the next annual meeting of the American Medical Association, namely: on the 3d of May, 1867. We would also respectfully suggest that all delegates to such convention be prepared to consider fully and act upon the following subjects:

*First.*—The adoption of a more uniform and just rate of lecture fees by all the colleges in this country.

*Second.*—The propriety of increasing the length of the annual lecture term and the number of professorships.

*Third.*—The adoption of measures for securing more thorough attention on the part of students, to the more elementary branches of medical science, and a more progressive order of medical studies.

*Fourth.*—The practicability of requiring three annual courses of lectures, instead of two, as a condition of graduation, and of making hospital clinical instruction a necessary part of the third course.

*Fifth.*—The practicability of establishing and exacting some appropriate standard of preliminary education for young men proposing to enter upon the study of medicine.

Feeling confident that a free interchange of views upon these and such other topics as the convention might deem proper, would result in the adoption of measures of great importance to the interests, honor, and usefulness of our profession, we again cordially and earnestly invite your coöperation.

N. S. Davis, S. D. Gross,
Worthington Hooker, M. Wright,
George C. Shattuck, *Committee.*

The medical colleges will, no doubt, be generally represented in this meeting, and as is suggested, an interchange of sentiment may be productive of good. It will be readily apparent that these subjects are not easily settled, and that what might be well, for one college, would not answer at all for another. The subjects are certainly, vital ones, and it is to be hoped that all medical colleges may be fully represented.

